# The Cervical‐Hyoid Dynamics in Correlation to Craniomandibular Disorders: A Report of Two Cases

**DOI:** 10.1155/crid/2044319

**Published:** 2026-09-15

**Authors:** Noureen Nahar, Atul P. Sattur, Megha Mary George, Krishna Burde, Jitendra Sharan, Smriti K. C. Basnyat

**Affiliations:** ^1^ Department of Oral Medicine and Radiology, Kalinga Institute of Dental Sciences and Hospital, Bhubaneswar, Orissa, India; ^2^ Department of Oral Medicine and Radiology, SDM College of Dental Sciences and Hospital, Dharward, Karnataka, India, sdmcds.org; ^3^ Department of Dentistry, All India Institute of Medical Sciences, Bhubaneswar, Orissa, India, aiims.edu; ^4^ Department of Prosthodontics, Kathmandu University School of Medical Sciences, Dhulikhel, Nepal, kusms.edu.np

**Keywords:** cervical vertebrae, hyoid bone, hyoid triangle analysis, neuromuscular orthotic, temporomandibular disorders

## Abstract

The hyoid bone maintains crucial anatomical and functional relationships with the mandible, pharynx, and cervical spine. Its position is dynamically influenced by surrounding musculature and fascial attachments. The spatial orientation of the hyoid bone plays a major role in muscular and joint imbalances in patients with temporomandibular joint disorders (TMJD). Neuromuscular orthotic therapy in patients with TMJDs can lead to measurable changes in hyoid bone positioning and cervical spine alignment. Using cone‐beam computed tomography (CBCT) and Rocabado′s hyoid triangle analysis, this case report presents two cases demonstrating repositioning of the hyoid bone through neuromuscular intervention, which promotes improved craniocervical alignment, correlating with functional changes in the joint position and postural modifications.

## 1. Introduction

Hyoid bone has its unique anatomic relationship in humans; it has no bony articulation but provides attachment to various muscles, ligaments, and fascia of the mandible, cranium, and pharynx [1]. It plays an important role in stabilization and optimal functioning of the stomatognathic system, along with cervical spine and the jaw. Studies have been done to determine the hyoid bone position in various facial pattern craniofacial hard tissue structures such as cervical vertebrae, cranial base, and jaw bones and the hyoid triangle assessment is one such method [1–4].

The hyoid triangle uses three cephalometric landmarks—retrognathion, hyoidale, and C3 cervical vertebrae—to assess the positional relationship between the mandible, hyoid bone, and cervical spine [1, 5]. This triangle is important for both prognosis and as a treatment indicator in correlating the effects of temporomandibular joint disorders (TMJDs) on craniocervical structures and also assessing the impact on the hyocervical complex. It has been noted that the relationship between the hyoid bone and the mandible is maintained from young ages, which correlates with the spurt of longitudinal growth in the cervical spine that, in turn, induces a vertical force to the mandible through the light elastic forces of the supra‐ and infrahyoid muscles [5]. Dentofacial morphology is closely interconnected with craniocervical posture and the position of the hyoid bone; both of these parameters are susceptible to alterations secondary to TMJ disorders [6]. Therefore, establishing a clear correlation between TMJ disorders, craniocervical posture, and hyoid bone positioning is essential. Understanding these relationships is critical not only for accurate diagnosis but also for determining the biomechanical and clinical consequences of TMJDs, thereby guiding prognosis and therapeutic interventions. The spatial orientation of the hyoid bone is not an independent factor but rather a developmental outcome of craniofacial growth and functional dynamics. Its position and biomechanical role are largely influenced by the vertical and anteroposterior growth of the maxilla and mandible, as well as by tongue posture and nasal respiration. When these developmental patterns are altered, such as in cases of chronic mouth breathing, maxillary narrowing, or neuromuscular imbalance, the hyoid bone tends to shift downward and rotate posteriorly, thereby heightening the risk of airway obstruction [7].

The primary objective of these case reports is to evaluate the spatial orientation of the hyoid bone before and after neuromuscular orthotic therapy in patients diagnosed with TMJD. The specific aims are as follows:

• To compare the position of the hyoid bone pre‐ and posttreatment utilizing cone‐beam computed tomography (CBCT).
• To assess modifications in Rocabado′s hyoid triangle for proper craniocervical and mandibular alignment.
• To investigate the correlation between the spatial relation of the hyoid bone and clinical symptomatology associated with TMJDs.

These case reports were prepared in accordance with the CARE guidelines to ensure completeness and transparency of clinical reporting.

## 2. Case Report

Clinical and radiological evaluation validates the diagnosis of two patients as internal derangement of the disc with or without reduction, along with craniomandibular instability and postural changes.

### 2.1. Case Report 1

#### 2.1.1. Diagnosis and Etiology

**Figure 1:**
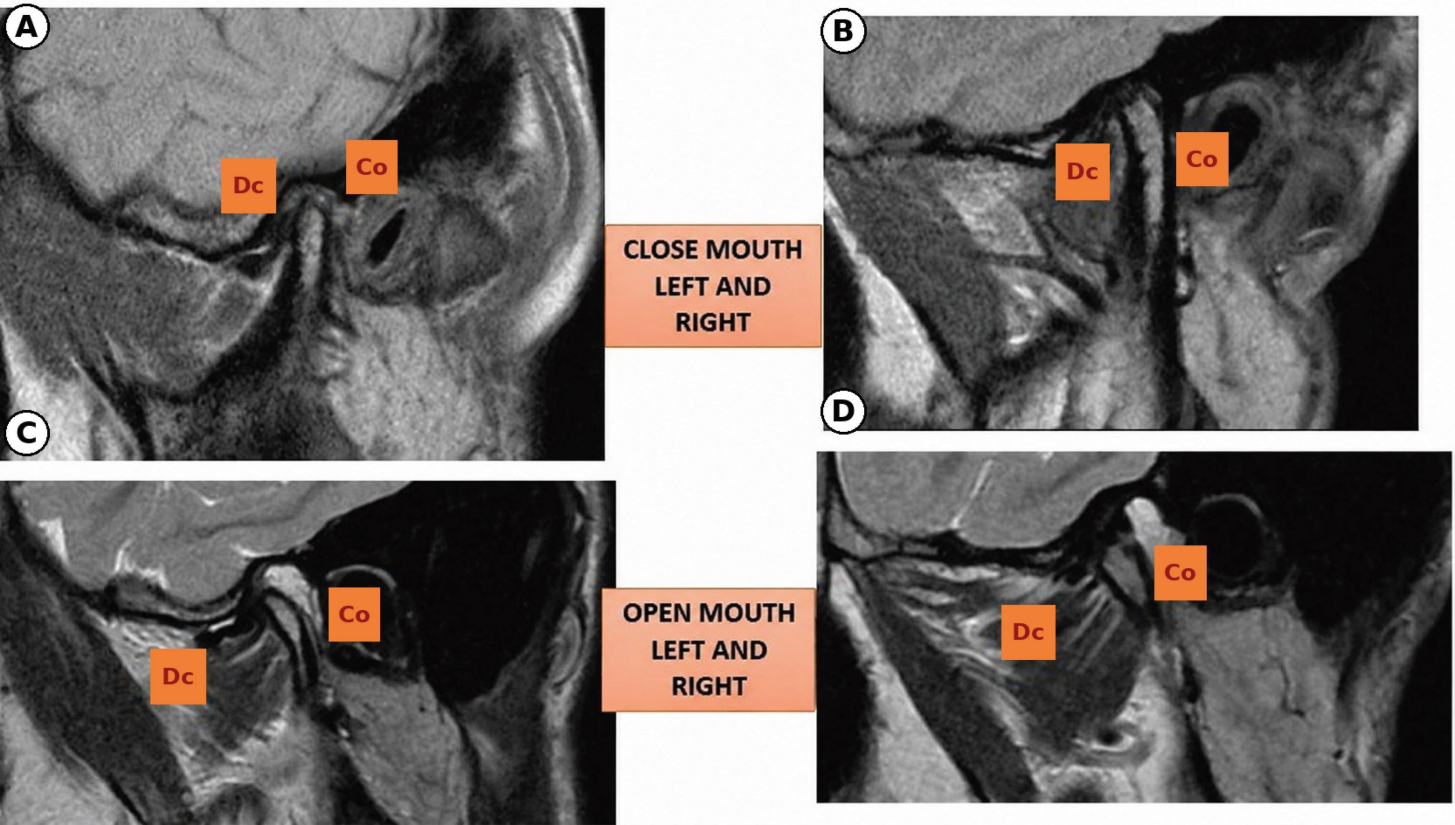
Sagittal magnetic resonance imaging (MRI) of the temporomandibular joints (TMJs) in Case 1, showing the right and left joints in closed‐mouth and open‐mouth positions. (A) Right TMJ, closed‐mouth position. (B) Left TMJ, closed‐mouth position. (C) Right TMJ, open‐mouth position. (D) Left TMJ, open‐mouth position. In each panel, Dc denotes the articular disc and Co denotes the mandibular condyle.

A 45‐year‐old male presented with bilateral TMJ pain, joint crepitus, restricted mouth opening, and bilateral headaches for 1 year. Clinical assessment was done using the Research Diagnostic Criteria for Temporomandibular Disorders (RDC/TMD criteria), indicating a provisional diagnosis of bilateral internal derangement without reduction [8]. Sophisticated imaging tools such as CBCT and magnetic resonance imaging (MRI) were done to know the true extent of TMDs before the commencement of any treatment. In both cases, CBCT was selected because a three‐dimensional assessment was required to evaluate osseous condylar morphology, joint space changes, and cervical‐hyoid relationships that could not be reliably assessed using conventional two‐dimensional radiography. CBCT was recorded in the natural head position, which revealed bilateral condylar beaking, flattening, articular space reduction, and decortication of the superior surfaces of bilateral joints. On the other hand, MRI was done in both open and closed‐mouth positions, which confirmed anterior disc displacement without reduction, accompanied by joint effusion, which was both confirmed by a 20 years experienced radiologist (Figure 1).

Laboratory tests have revealed the alterations in blood chemistry and general health, indicating a nutritional deficit (Table 1). The laboratory investigations were performed to exclude systemic or metabolic factors that could contribute to musculoskeletal pain and to optimize overall patient management. In the biometric evaluation, neuromuscular analysis showed a positive Learreta test, which is measured using the EMG response of the masticatory muscles at maximal occlusion in four different occlusal vertical dimension (OVD) positions in such a way that the joint undergoes decompression, and subsequently, relief of the inhibitory motor response of trigeminal nerve, following the test, along with joint vibration analysis (JVA), diagnosed both joints as Piper Stage 4b, indicating chronic disc displacement at the medial pole of condyle with ongoing functional adaptation, as distinct from earlier, more acute Piper stages [9, 10].

**Table 1:** Laboratory values highlight the alterations in blood chemistry, indicating a nutritional deficit state.

Parameters	Patient value	Reference range
Hemoglobin (Hb)	12 g/dL	Male: 13.8–17.2 g/dL
		Female: 12.1–15.1 g/dL
Vitamin D	10 ng/ml	30–100 ng/mL
Vitamin B12	170 pg/ml	200–900 pg/mL
Peripheral blood smear	Normal	Normal

### 2.2. Case Report 2

#### 2.2.1. Diagnosis and Etiology

A 28‐year‐old male presented with a history of bilateral joint clicking, intermittent pain, normal mouth opening with pain in wide opening, and headaches for 2 years. Clinical assessment was done using the RDC/TMD criteria, giving a provisional diagnosis of bilateral internal derangement without reduction [8].

**Figure 2:**
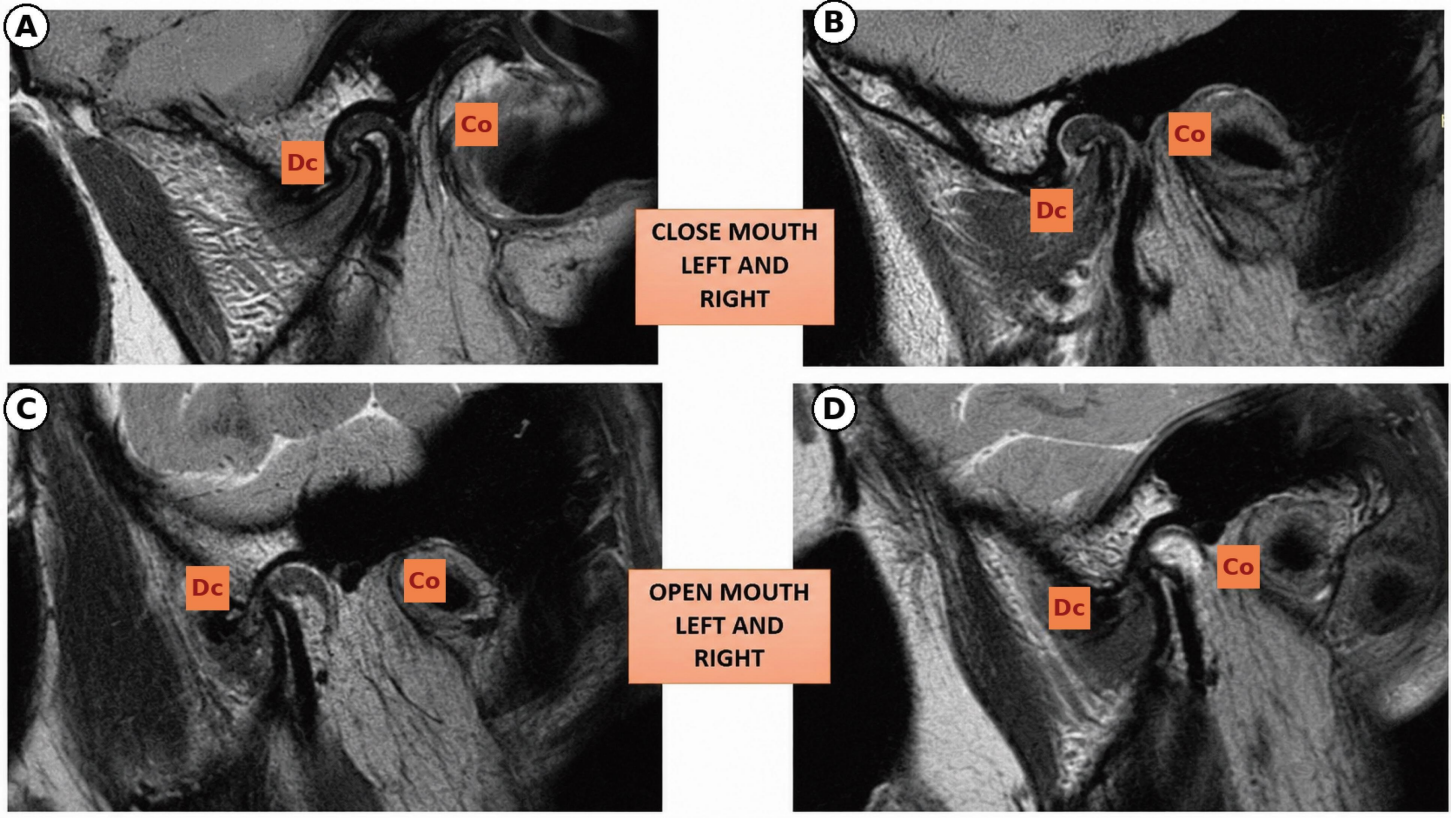
Sagittal magnetic resonance imaging (MRI) of the temporomandibular joints (TMJs) in Case 2, showing the right and left joints in closed‐mouth and open‐mouth positions. (A) Right TMJ, closed‐mouth position. (B) Left TMJ, closed‐mouth position. (C) Right TMJ, open‐mouth position. (D) Left TMJ, open‐mouth position. In each panel, Dc denotes the articular disc and Co denotes the mandibular condyle.

Pretreatment CBCT was recorded in the natural head position, revealing bilateral beaking of condyles, flattening, articular space reduction, and decortication of the superior surfaces of the bilateral joints. MRI was done in both open and closed mouth, confirming bilateral anterior disc displacement without reduction, which was both confirmed by a 20 years experienced radiologist (Figure 2).

In the biometric evaluation, neuromuscular analysis showed a positive Learreta test, which is measured using the EMG response of the masticatory muscles at maximal occlusion in four different OVDs in such a way that the joint undergoes decompression, and subsequently, relief of the inhibitory motor response of trigeminal nerve, following the test, along with JVA, diagnosed both joints as Piper Stage 4b and 5a. Stage 4b denotes chronic nonreducing disc displacement at the medial pole with ongoing functional adaptation, whereas Stage 5a denotes an actively painful perforation of retrodiscal tissue, consistent with internal displacement of disc without reduction and adapting osteoarthritic changes [9, 10].

### 2.3. Treatment Outcomes

Follow‐up CBCT was obtained after 1 year of treatment to assess changes in hyoid position, cervical alignment, and condylar relationship following neuromuscular orthotic therapy. The anticipated diagnostic benefit was considered to outweigh the radiation exposure, as both scans directly influenced treatment assessment. Both patients were managed with a standardized treatment protocol. The protocol involves the fabrication of a neuromuscular orthotic, individualized using each patient′s biometric measurements, and is prescribed for continuous wear (24 h/day), with monthly calibration of the orthotic using biometric instruments and T‐scan for proper craniomandibular stability. In a physiologically balanced craniocervical relationship, Bibby and Preston′s (Rocabado′s) hyoid triangle is typically characterized by an inferiorly directed apex, with the hyoid bone positioned at or near the level of the third cervical vertebra (C3) [1, 5]. In contrast, a hypodivergent configuration, with the hyoid bone displaced superiorly and anteriorly to this level, as observed on the pretreatment imaging of both cases presented below, is considered indicative of altered hyomandibular and craniocervical balance [1, 5].

**Figure 3:**
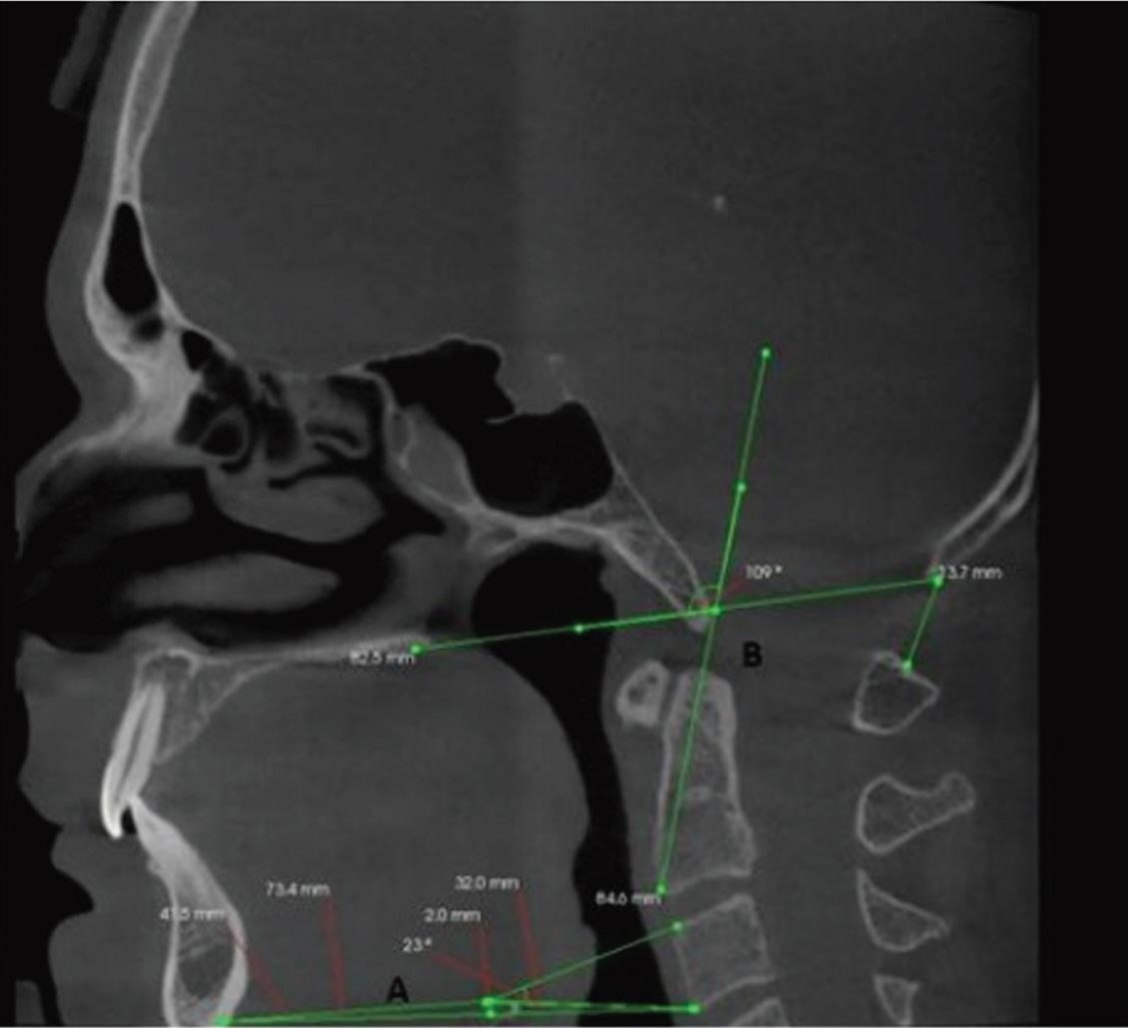
Case 1 pretreatment CBCT reveals a hypodivergent hyoid triangle. (A) The hypodivergent triangle with an angulation of 23 degrees. (B) Cervical vertebrae alignment.

**Figure 4:**
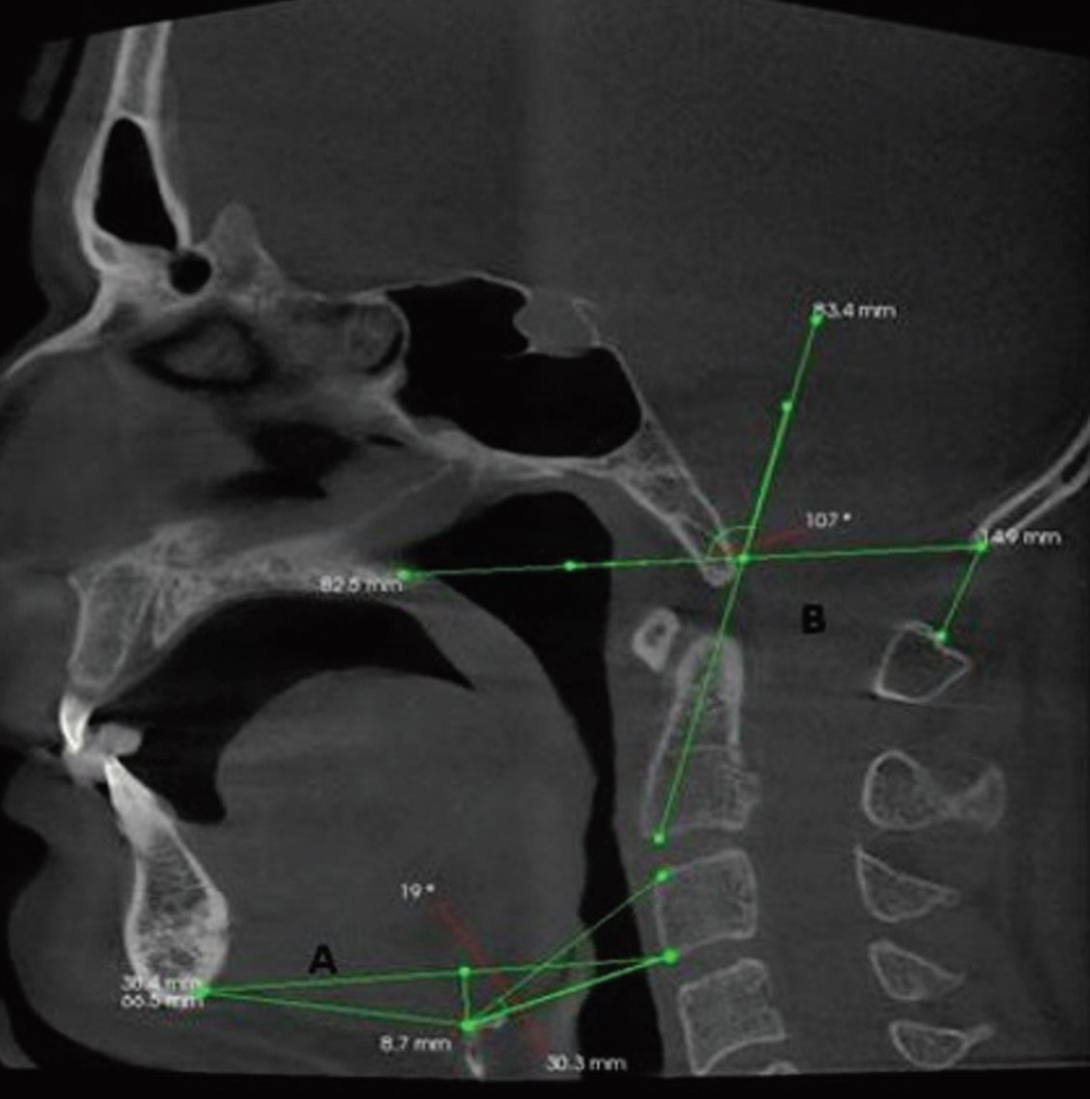
Posttreatment CBCT of Case 1 reveals the hyoid bone at the level of C3 vertebrae. (A) An upward‐facing triangle with an angulation of 19 degrees. (B) Cervical vertebrae alignment.

In Case 1, a similar treatment protocol was followed with both pre‐ and posttreatment CBCT, revealing a narrow hypodivergent hyoid triangle (Rocabado′s hyoid triangle analysis [1, 5]) with the hyoid bone positioned below C3. The pretreatment angle between the hyoid bone and cervical spine was measured to be 23° (Figure 3). After 1 year of continuous orthotic use, the posttreatment CBCT revealed the greater cornua of the hyoid bone aligned at the level of C3, restoration of normal cervical curvature, and an inverted hyoid triangle apex pointing toward the floor. The angle between the hyoid bone and cervical spine decreased to 19°, indicating improved spatial orientation of the hyoid bone and craniocervical balance (Figure 4).

**Figure 5:**
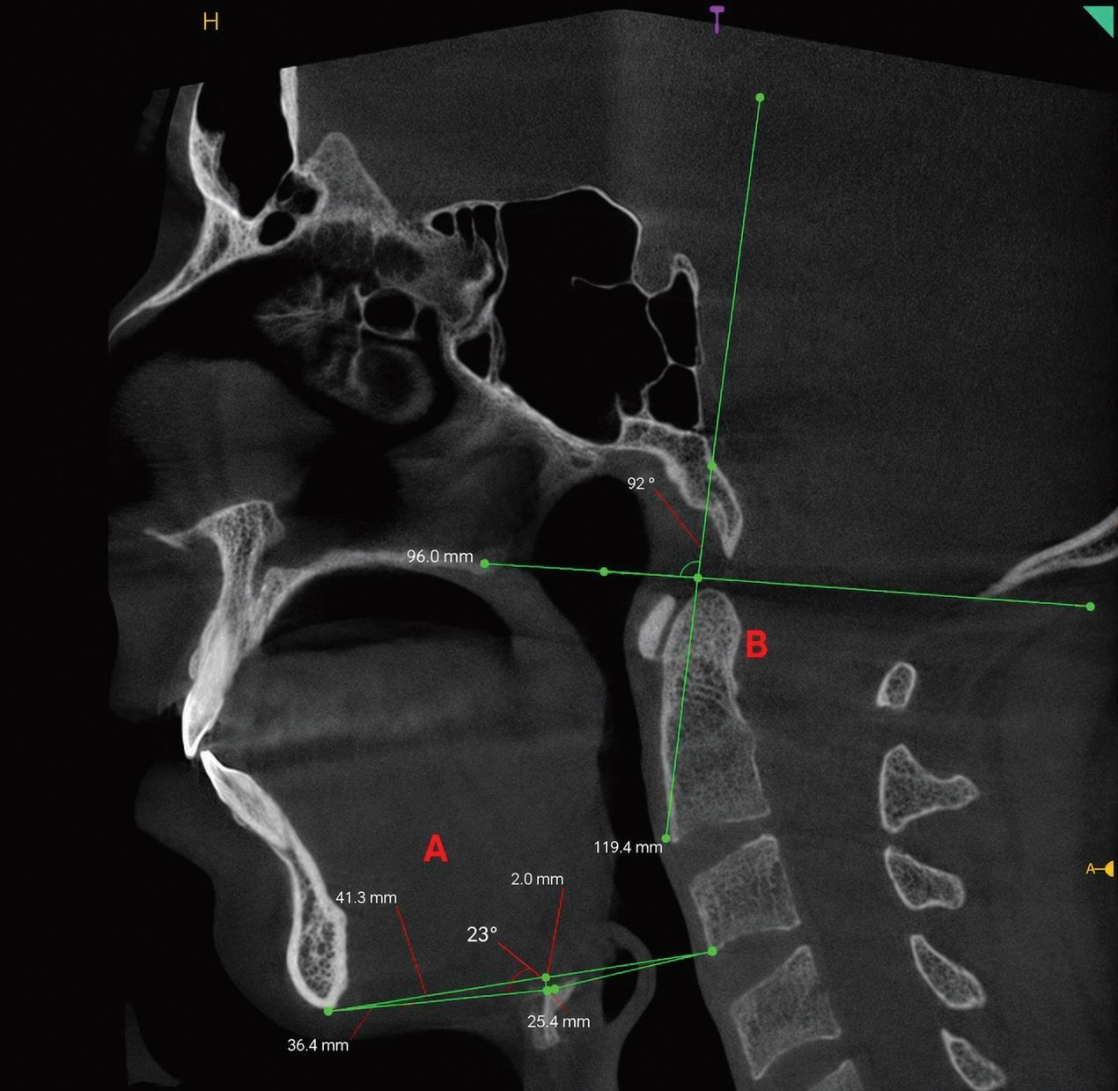
Case 2 pretreatment CBCT reveals a narrow hypodivergent hyoid triangle. (A) A hypodivergent triangle placed with the hyoid bone below the level of C3 with an angulation of 23 degrees. (B) Cervical vertebrae alignment.

**Figure 6:**
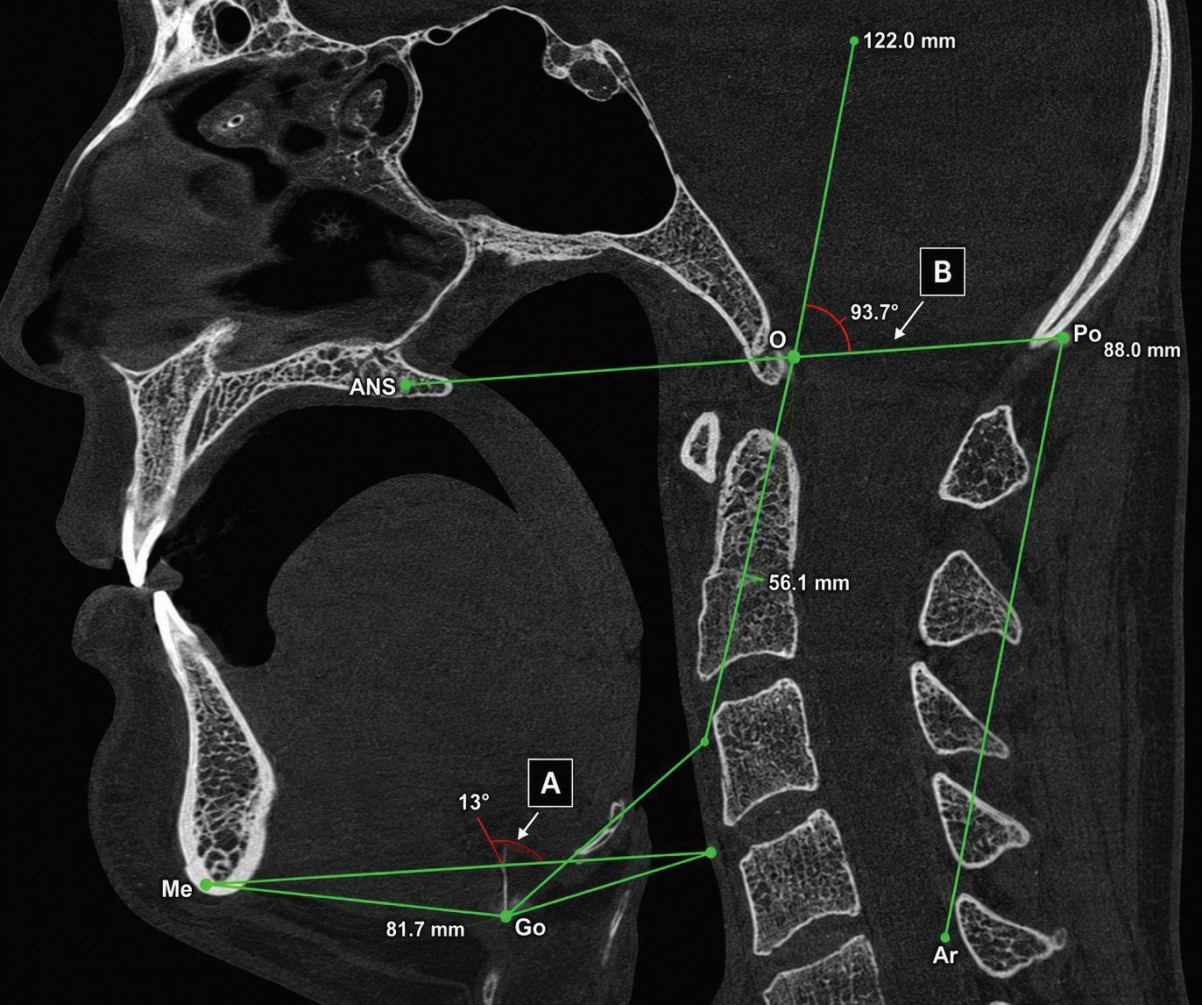
Posttreatment CBCT of Case 2. (A) A hypodivergent hyoid triangle with the hyoid bone placed at C3 with an angulation of 19 degrees (B) Cervical vertebrae alignment.

In Case 2, the patient followed the same treatment protocol as Case 1. Pretreatment CBCT revealed a hypodivergent hyoid triangle (Rocabado′s hyoid triangle analysis [1, 5]) with the hyoid bone positioned below the level of C3. After 1 year of continuous orthotic wear, posttreatment imaging demonstrated a triangle with apex pointing downwards and hyoid bone position at the level of C3, consistent with improved biomechanical alignment and muscle function (Figures 5 and 6).

Across both cases, posttreatment CBCT demonstrated improved spatial orientation of the hyoid bone, restoration of cervical curvature, and improved craniocervical alignment following 1 year of neuromuscular orthotic therapy.

## 3. Discussion

TMJDs or craniomandibular disorders often affect the masticatory muscles and related structures. In this study, patients presented with typical TMD symptoms and underwent pre‐ and posttreatment CBCT and MRI assessments, along with biometric evaluation. TMJ internal derangement in the present cases was classified using the RDC/TMD clinical criteria in conjunction with MRI findings and the Piper/JVA classification. The Piper classification was selected because it is applicable to the diagnosis and conservative management of TMJ internal derangement, whereas the Wilkes classification is primarily intended for staging disease progression and guiding surgical management. Bibby′s hyoid triangle, formed by retrognathion, hyoidale, and C3, was designed to minimize postural variations in cephalometric analysis [1]. Although the hyoid bone is not routinely evaluated in dental practice, its spatial orientation possesses a significant effect on anatomical and functional relations to the TMJ and cervical vertebrae. It maintains a stable relationship with the C3, making it a reliable reference point in cephalometric analysis as noted by Rocabado [5].

The hyoid also follows mandibular motion during cervical flexion–extension, reinforcing its relevance in postural assessments. Although lateral cephalograms have limitations due to 2D radiograph and overlapping structures, CBCT provides accurate 3D visualization, making it ideal for evaluating hyoid positioning. The resting position of the hyoid bone, which is devoid of bony articulation, is maintained by the tension equilibrium between the suprahyoid and infrahyoid musculature, along with the position of the mandible [6].

Rocabado′s hyoid triangle leverages planes extending between the mandibular symphysis and cervical vertebrae, thereby minimizing the effect of changes in head posture and eliminating the need for cranial reference planes [5].

Clinically, inferior movement of the hyoid bone is significant, as it contributes to improved airway patency by increasing the oropharyngeal space [6]. At the initiation of treatment, the patients in the present case reports exhibited symptoms of TMDs along with a superiorly positioned hyoid bone. Following neuromuscular orthotic therapy, the hyoid bone was repositioned to a more inferior and posterior orientation, corresponding with symptom resolution and a more favorable craniocervical alignment. An elevated hyoid bone position is associated with a reduction in the posterior lingual airway space, which can have functional implications for airway dynamics [11]. Although the posttreatment CBCT suggested an apparent change in the visible oropharyngeal airway, dedicated volumetric airway analysis was beyond the scope of this report. As the CBCT examinations were performed primarily to assess temporomandibular joint structures and hyoid position, no definitive conclusions regarding airway changes can be drawn from the present findings.

The posttreatment findings in both cases suggest that neuromuscular orthotic therapy not only repositions the mandible and improves TMJ function but also facilitates a biomechanically favorable change in hyoid bone position, contributing to overall structural and symptomatic improvement. The present observations are consistent with the findings of Sreenivasan et al. [6], who demonstrated improvement in hyoid positioning following TMJ management using CBCT. Similarly, Pettit and Auvenshine [11] reported favorable changes in hyoid position following successful management of myofascial pain. The current report extends these observations by demonstrating corresponding changes in Rocabado′s hyoid triangle following neuromuscular orthotic therapy.

Because cervical posture influences hyoid position and craniocervical biomechanics, routine clinical evaluation of cervical alignment—including assessment of the C1–C7 axis and forward head posture—may provide valuable adjunctive information in patients with TMD. Future studies should investigate whether cervical stabilization exercises and postural correction can complement radiographic assessment and improve treatment outcomes.

## 4. Conclusions

This case report highlights the significance of hyoid bone positioning as a biomechanical marker in patients with TMJDs. The use of neuromuscular orthotic therapy may result in favorable postural and anatomical changes, as evidenced by CBCT imaging and Rocabado′s hyoid triangle analysis. Both patients demonstrated a shift of the hyoid bone from a superior and anterior position to a more physiologic alignment at the level of the C3, accompanied by improved craniocervical posture and symptomatic relief. These findings support the role of neuromuscular intervention in restoring functional balance within the stomatognathic system and suggest that hyoid bone assessment may serve as a valuable adjunct in the diagnosis and management of TMD. Further studies with larger cohorts are recommended to validate these observations.

## 5. Limitations

This report is limited by its descriptive nature and the inclusion of only two cases, which precludes generalization of the findings to the broader TMJD population. As a retrospective case report without a control group, the observed changes in hyoid position and craniocervical alignment should be interpreted as associations rather than evidence of a causal relationship with neuromuscular orthotic therapy. Although the pre‐ and posttreatment CBCT scans were acquired using the same imaging protocol with patients in the natural head position, exact reproduction of an identical midsagittal reference plane between serial scans cannot be guaranteed. Minor variations in head position, mandibular posture, or slice selection may have influenced the linear and angular measurements of the hyoid triangle. In addition, the follow‐up period was limited to 1 year; therefore, the long‐term stability of the observed radiographic changes remains uncertain. Larger prospective studies with standardized imaging protocols, longer follow‐up, and objective clinical outcome measures are required to validate these preliminary observations.

## Author Contributions

**Noureen Nahar:** conceptualization, methodology, investigation, data curation, formal analysis, visualization, writing – original draft, writing – review and editing. **Atul P. Sattur:** conceptualization, supervision, methodology, writing – review and editing. **Megha Mary George:** investigation, validation, writing – review and editing. **Krishna Burde:** methodology, validation, writing – review and editing. **Jitendra Sharan:** supervision, resources, writing – review and editing. **Smriti KC Basnyat:** investigation, validation, writing – review and editing.

## Funding

No funding was received for this manuscript.

## Disclosure

All authors have read and approved the final version of the manuscript and agree to be accountable for all aspects of the work.

## Ethics Statement

Formal ethical approval was not required for this case report because it involved the retrospective use of existing clinical records and imaging data (CBCT and MRI) acquired as part of the patients′ routine diagnostic evaluation and treatment. No additional investigations or interventions were performed for research purposes. All patient identifiers were removed, and no extraoral photographs or other identifiable information are included in this report.

## Consent

Informed consent was obtained from the patient in accordance with ethical principles, granting permission for the use and publication of their medical data, images, and case details in this report. The patient′s information has been anonymized to protect privacy and is intended for educational and research purposes.

## Conflicts of Interest

The authors declare no conflicts of interest.

## Supporting information


**Supporting Information** Additional supporting information can be found online in the Supporting Information section. Supporting Information. File S1: CARE checklist of information to include when writing a case report.

## Data Availability

Data are available on request from the authors.
